# *In vitro* study of spontaneous motility and cholinergic responses in the human Hirschsprung's disease colon

**DOI:** 10.3389/fped.2025.1698220

**Published:** 2025-12-01

**Authors:** Felix Schulze, Judith Lindert, Rüdiger Köhling, Stefanie Märzheuser, Timo Kirschstein

**Affiliations:** 1 Department of Paediatric Surgery, University of Rostock, Rostock, Germany; 2 Oscar Langendorff Institute of Physiology, University of Rostock, Rostock, Germany; 3 Center of Transdisciplinary Neurosciences Rostock (CTNR), University of Rostock, Rostock, Germany

**Keywords:** Hirschsprung's disease, organ bath, carbachol, atropine, contractility, colon, aganglionosis

## Abstract

**Background:**

The current standard treatment of Hirschsprung's disease (HD) involves complete resection of the aganglionic intestinal segment with routine rectal irrigation pre- and postoperatively. To date, our understanding of the underlying pathomechanisms is not sufficient to warrant modifying this therapeutic approach. Here, we utilized resected tissue specimens from children with HD to investigate spontaneous motility and cholinergic responses in circular muscle from both ganglionic and aganglionic colon *in vitro*.

**Methods:**

Full-thickness intestinal samples (from both aganglionic and ganglionic segments) were obtained intraoperatively from children with HD undergoing pull-through surgery. After removing the mucosal tissue and longitudinal smooth muscle, we prepared specimens consisting of circular muscle layer and submucosal tissue. These deafferentiated specimens were mounted in the organ bath to record isometric forces during spontaneous motility or following cholinergic stimulation.

**Results:**

Regarding spontaneous motility, the basal tone was observed in all specimens, and phasic contractions were observed in 81% vs. 63% of ganglionic and aganglionic specimens, respectively. Both basal tone and phasic contractions were abolished in the presence of 40 mM EDTA indicating Ca^2+^ dependence, yet atropine (1 µM) had no effect on these measures. Increasing concentrations of the muscarinic receptor agonist carbachol raised the basal tone of ganglionic specimens, but significantly less potently in aganglionic tissue. In contrast, carbachol-evoked phasic contractions were still observed and even larger in aganglionic specimens than in ganglionic tissue.

**Conclusions:**

We conclude that the circular muscle of aganglionic colonic segments retains the ability to establish a Ca^2+^-dependent and atropine-insensitive spontaneous motility similar to the circular muscle of ganglionic tissue. However, both tissue segments differed considerably in response to external cholinergic stimulation with significantly enhanced phasic-to-tonic contraction ratios in aganglionic specimens. These findings will help understand the persistent circular muscle contraction in aganglionic bowel which is still a major challenge in the preoperative HD management.

## 1 Introduction

Hirschsprung's disease (HD) is a rare malformation of the gastrointestinal tract, affecting 1/5,000 live births with a male preponderance (4:1) (1). The pathogenesis of this malformation has not yet been conclusively clarified (2). Non-syndromic HD has been attributed to a dysregulation of the Sox10 gene and the RET gene (3). It is believed that HD is a migration disorder of intra-intestinal ganglion cells from the oral to the aboral direction during neural crest development, leading to a permanent contraction of the affected intestinal segment proximal to the anal canal as a result of aganglionosis (4, 5). The extent towards oral direction is variable, but is confined to the rectosigmoidal colon in approx. 80%–85% of all cases (6). Meanwhile, complete aganglionosis of the large intestine [total colonic aganglionosis (TCA)] and even of the large and small intestine are regarded as independent in pathogenesis and therapy (7, 8). The malformation leads to delayed meconium passage, persistent constipation and abdominal distension (9). At present, the primary treatment is the complete removal of the aganglionic bowel segment (10). Nevertheless, diagnosis and treatment are still challenging due to the variability of the malformation and misinterpretation of the medical history and clinical findings (11, 12). In addition to constipation and failure to thrive, severely affected patients suffer from comorbidities either resulting from HD or occurring independently (13), which may be lethal in 1%–10% of all cases (14).

The pathophysiological hallmark is the distal stenosis in the aganglionic colon, which is believed to be caused by the persistent contraction by the circular smooth muscle layer in the affected colon. Our understanding of the pathophysiology, however, is highly insufficient. It mainly suffers from the lack of adequate animal models; and evidence from human tissue is scarce. On the one hand, it is known that acetylcholine levels in aganglionic muscle are about three times higher than those in ganglionic tissue (15). On the other hand, aganglionic circular muscle showed reduced acetylcholine-induced responses (16, 17), while aganglionic longitudinal (taenial) muscle once had normal cholinergic responses (18), but also showed less responsivity to (19) or even a higher sensitivity to acetylcholine (20).

Taken together, there is an inconsistent concept of cholinergic responsivity of HD smooth muscle. Moreover, spontaneous phasic contractions were not observed in longitudinal aganglionic muscle strips (19). Here, we directly compared aganglionic and ganglionic tissue from patients with HD consisting of circular muscle and *tela submucosa* to study spontaneous motility and cholinergic responses in a paired fashion. We found that spontaneous motility consisting of a basal tone and phasic contractions were present in aganglionic circular muscle, but cholinergic responses were differentially altered in this tissue.

## 2 Methods

### 2.1 Human Hirschsprung's disease samples

Patients with Hirschsprung's disease (HD) with and without a stoma underwent daily rectal irrigation (21) before the pull-through surgery at the Department of Paediatric Surgery. The diagnosis of Hirschsprung's disease was confirmed prior to the procedure by a rectal biopsy, either at the patient's local hospital or at our clinic. When a stoma was present and this stoma had to be anastomosed intraoperatively, the stool was preoperatively transferred once a day from the oral stoma to the aboral stoma. The reason for this procedure, which is part of our clinical practice, is based on clinical evidence that the aboral parts of the intestine, i.e., those located distal to the stoma, were faster to regain function after anastomosis if stool had been transferred beforehand. All patients with HD received a preoperative colon contrast enema and intestinal biopsies to confirm the diagnosis. A transanal pull-through procedure with full-wall preparation from 2 cm from the dentate line (22) was performed. From the pull-through resectate, one part was taken from the aganglionic segment and one part from the ganglionic segment confirmed by intraoperative frozen section biopsy (23, 24). The ganglionic sample for organ bath measurements was always taken orally from the ganglionosis confirmed by the frozen section, and the aganglionic sample was always taken as distally as possible, noting that in the de la Torre pull-through surgery performed at our institution, the first 1–2 cm are not prepared transmurally. The resected intestinal segments were examined postoperatively at the level of the sample collection for our study by our experienced pathologists using staining methods to detect the presence of aganglionosis.

**Figure 1:**
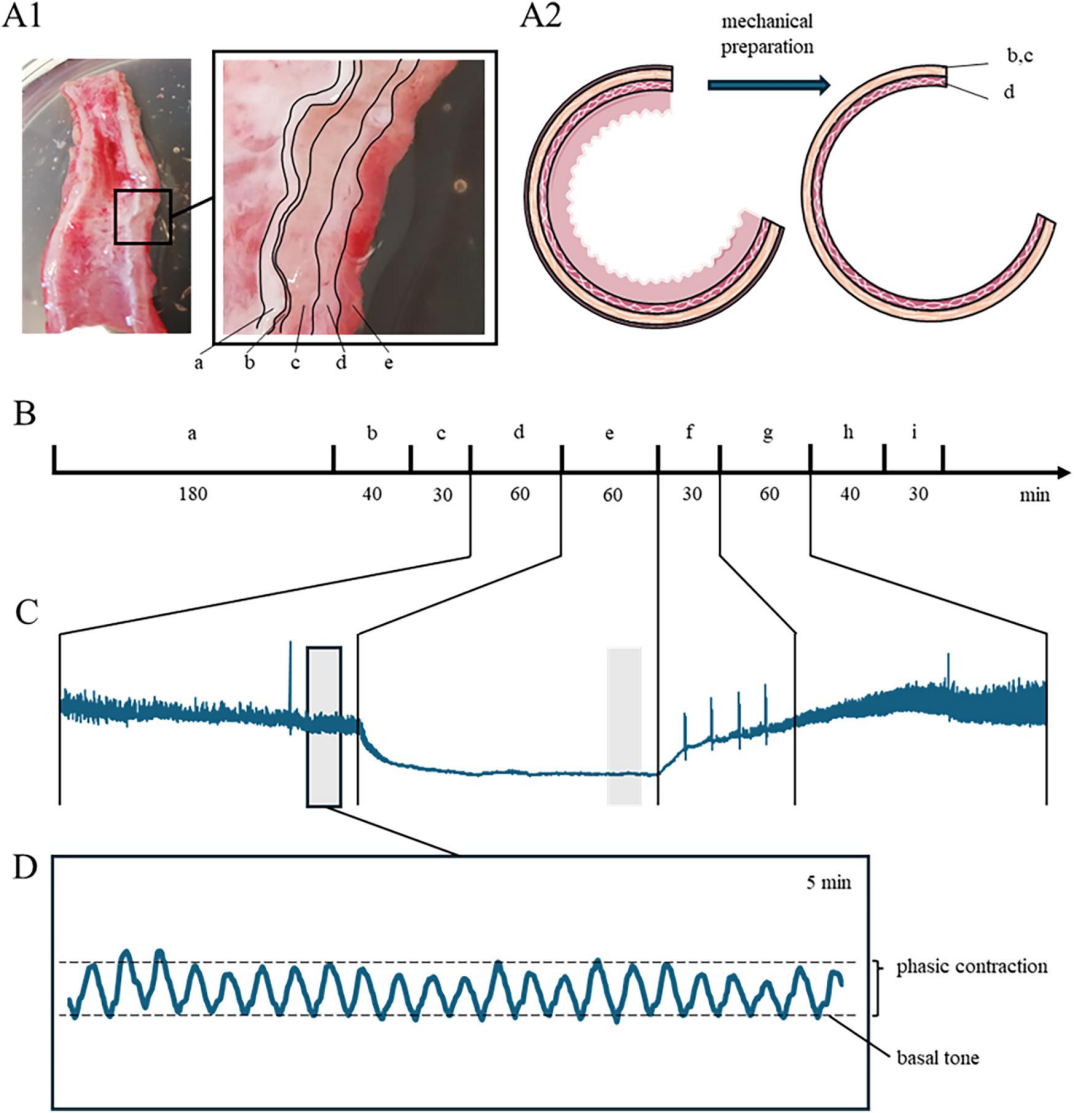
**(A1)** Full wall preparation of a surgical resectate, the wall layering is shown in the magnification: (a) serosa, (b) longitudinal muscularis, (c) circular muscularis, **(d)** tela submucosa, (e) mucosa. **(A2)** The serosa and the mucosa, with the exception of the tela submucosa, were removed from the preparation. The muscle strips were then dissected in a circular muscle direction. **(B)** Timeline (in min): After the insertion into the organ bath, the specimens were allowed to equilibrate.: Equilibration (a) is followed by induced contraction by KCl (b), followed by a washout phase (c). After another equilibration (d), the test compound (EDTA, atropine, carbachol) is added (e), followed by another washout phase (f) and equilibration (g). The final step is another induced contraction with potassium chloride (h) with a subsequent washout phase (i). **(C)** Enlargement of section d to g (from panel B). Unless otherwise stated, the force data were taken at the time points indicated by gray boxes. **(D)** The basal tone was defined as the mean value of all local minima, the amplitude of the phasic contractions was defined as the difference between the mean values of the local maxima and minima.

Both ganglionic and aganglionic parts were obtained intraoperatively, rapidly stored in a pre-oxygenized preparation solution containing (in mM) 120 NaCl, 4.5 KCl, 26 NaHCO_3_, 1.2 NaH_2_PO_4_, 1.6 CaCl_2_, 1.0 MgSO_4_, 0.025 Na_2_-EDTA, 5.5 glucose, 5 HEPES (pH = 7.4) at 4°C, and transferred to the Institute of Physiology. From these parts, both serosal and mucosal tissue were removed, and specimens for the organ bath (size 3 × 12 mm, including circular muscle and submucosal tissue) were prepared in fresh preparation solution of the above composition at 4 °C (Figure 1A). During mechanical removal of the mucosa, care was taken to preserve the *lamina muscularis mucosae* as a separating layer from the *tela submucosa*. We obtained 8 specimens from each patient (4 from ganglionic and 4 from aganglionic tissue, respectively).

The total population (enrolled between March 2023 and June 2024) consisted of eight patients who underwent pull-through surgery. Inclusion criteria were the absence of infections or circulatory disorders in the affected bowel segments and appropriate information and consent was taken from their legal guardians. The patients' characteristics are given in Table 1. The study was approved by the local Ethics Committee (approval no. A2022-0177).

**Table 1:** Patients' characteristics. None of the patients underwent multiple operations during the study. Patients 3 and 4 received their stomas prior to admission to our department.

Patient	Sex	Age at surgery (months)	Body height (cm)	Body weight (kg)	Resectate length (cm)	Transition zone	Stoma
1	Male	37	88	16.6	11	Rectosigmoid	None
2	Female	6	65	7.3	9.5	Rectosigmoid	None
3	Female	20	80	8.9	17	Rectosigmoid	Ileostoma
4	Male	5	75	8.5	17	Rectosigmoid	Ileostoma
5	Male	5	65	8	10	Rectosigmoid	None
6	Male	4	62	5.3	15	Rectosigmoid	None
7	Male	20	82	10.5	24	Rectosigmoid	None
8	Male	7	75	10.6	8	Rectosigmoid	None

### 2.2 Organ bath measurements

The muscle strips were gently fixed at their ends with threads and transferred to an organ bath chamber (Automatic Organ Bath, Panlab, Harvard Apparatus, Holliston, Massachusetts, USA) with a buffer of the following composition (in mM): 120 NaCl, 28 NaHCO_3_, 4.7 KCl, 2.0 sodium pyruvate, 2.5 CaCl_2_, 1.2 MgCl_2_, 0.5 Na_2_-EDTA and 5.5 D-glucose (95% O_2_, 5% CO_2_; pH 7.4; osmolality 306-314 mosmol/kg H_2_O). The organ bath (25 mL volume) was heated to 37°C (LE 13206, Letica Scientific Instruments, Vendenheim, France). The lower end of the specimen was fixed to the bottom of the chamber via the thread, the upper end was coupled to a calibrated force transducer (Panlab MLT0201) connected to a computer via an amplifier (Quad Bridge Amp, AD Instruments, Spechbach, Germany) and a recording unit (PowerLab 8/30 and LabChart 7 software, AD Instruments). The specimens were preloaded with a tension of 2–3 mN. All recordings began after a 3 h equilibration period, as preliminary tests to establish and validate the experimental protocol had shown that spontaneous contractions if they occurred, they did so after a 3 h equilibration period at the latest (see Supplementary Figure S1). To standardize the protocol, the same procedure was used for all samples. All experiments began with an initial KCl pre-test (period “b” in Figure 1B; adding 1 mL of 1.5 M stock solution) to confirm depolarization-induced contraction (25). The whole experimental work-up is shown in Figure 1B.

In the first set of experiments, we evaluated the spontaneous basal tone as well as phasic contractions present after the equilibration time. Then, both the Ca^2+^ dependence and the muscarinic receptor contribution were assessed by adding 0.5 mL of 2 M ethylene diamine tetraacetic acid [EDTA (Sigma, E1644)] final concentration 40 mM) or 100 µL of 0.25 mM atropine (Merck, 1.59508) (final concentration 1 µM), respectively, for 60 min to the buffer (period “e” in Figure 1B). The basal tone was determined as the arithmetic mean of all local force minima within a time frame of 5 min (Figure 1C) 10 min prior to (black-lined gray box in Figure 1C) and 50 min after application of EDTA or atropine (gray box in Figure 1C). The amplitude of phasic contractions was determined as the difference from the basal tone to the arithmetic mean of all local force maxima within the same time frame (Figure 1D). To prevent interference between EDTA or atropine, a given specimen was tested with EDTA or atropine only. Time control experiments were carried out using vehicle (100 µL of distilled water).

In the second set of experiments, we tested the specimen's responsivity to external cholinergic stimulation using a step-wise increase of the carbachol concentration. To this end, we added 100 µL of increasing carbachol (Tocris, 2810) stock solutions (from 2.5 µM to 25 mM) to the buffer (volume 25 mL) every 10 min without washout to yield final concentrations of 0.01–100 µM (during period “e” in Figure 1B). Carbachol-evoked tonic and phasic contraction amplitudes were determined as described above (Figure 1C). In addition, we calculated the phasic-to-tonic contraction ratio to normalize the propensity to produce phasic contractions to its respective tone for each specimen. At the end of each experiment, we repeated the KCl application to confirm the preserved depolarization-induced contraction. Comparing the KCl response at the end of the experiment with the response at the beginning served to validate stable recording conditions (25).

### 2.3 Statistics

Unless otherwise indicated, data were expressed as mean values ± the standard error of the mean (SEM). For statistical comparisons, data were tested for normal distribution, and then analyses were performed using the appropriate test as indicated. The level of significance was set to *P* < 0.05, and significant differences were indicated with asterisks in all figures (*P < 0.05, **P < 0.01, ***P < 0.001).

## 3 Results

### 3.1 Occurrence of spontaneous contractions

The first aim of the present study was to compare ganglionic and aganglionic colonic specimens regarding their spontaneous motility which consisted of a basal tone and phasic contractions. The basal tone developed after 3 h of equilibration, plateaued at values around 4 mN and was significantly different for both tissue types (ganglionic: 4.64 ± 0.57 mN; aganglionic: 3.54 ± 0.62 mN, *P* < 0.05, Mann–Whitney). By the end of this equilibration period, phasic contractions riding on this basal tone had developed in 26/32 ganglionic and 20/32 aganglionic specimens (*P* = 0.095, *χ*^2^ test). In addition, they were significantly smaller in aganglionic (1.18 ± 1.67 mN, *n* = 20) than in ganglionic tissue (5.91 ± 1.70 mN, *n* = 26, *P* < 0.001, Mann–Whitney test). Challenging the circular muscle with high KCl produced significantly higher tonic contraction in ganglionic than aganglionic specimens (ganglionic: 16.4 ± 1.4 mN, *n* = 32; aganglionic: 9.7 ± 1.1 mN, *n* = 32; *P* < 0.001, *t*-test). Intriguingly, following high KCl administration, all specimens from both groups presented phasic contractions (see Supplementary Figure S2).

### 3.2 Effects of EDTA and atropine on phasic contractions

**Figure 2:**
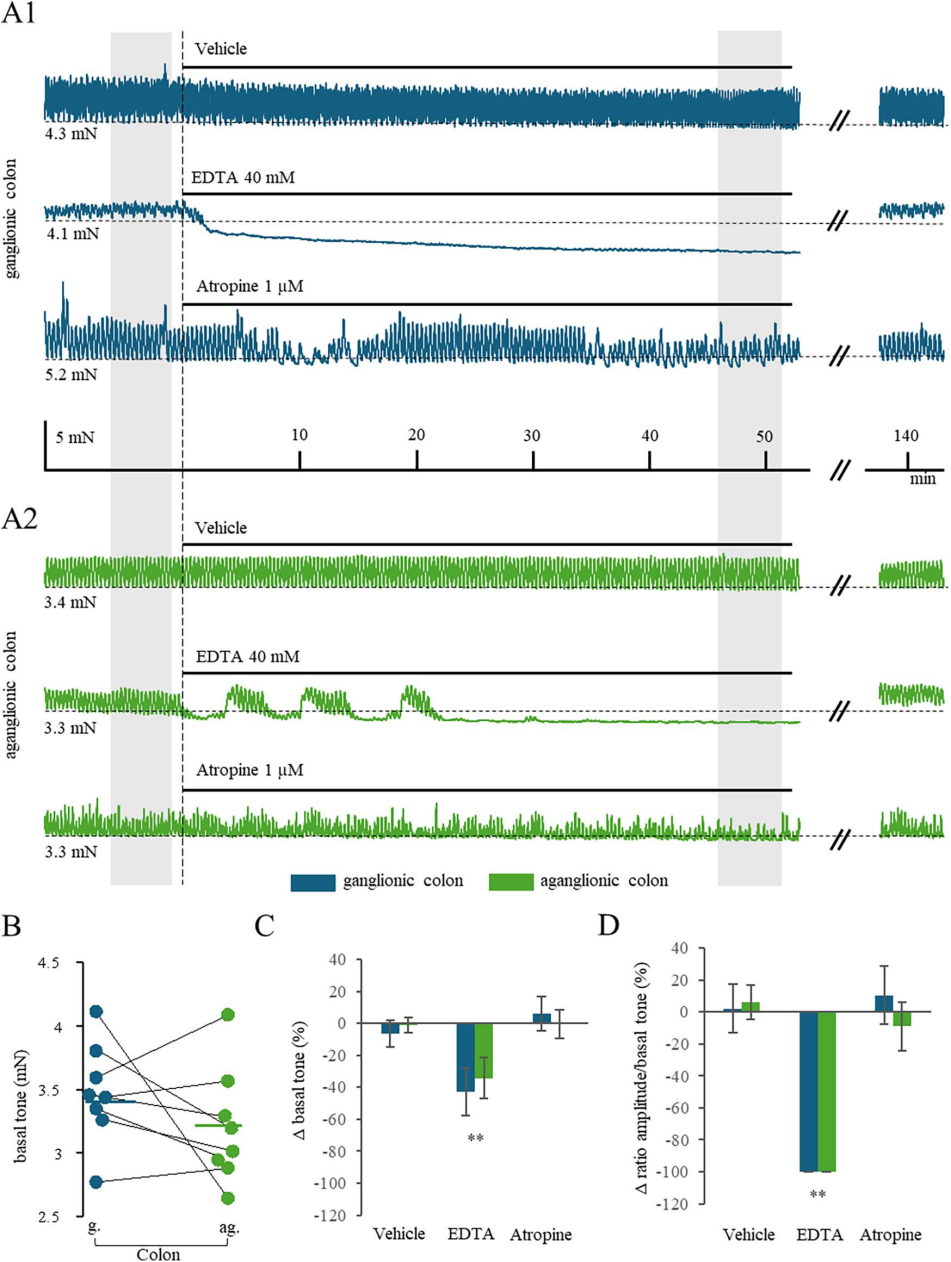
**(A)** Example traces of three samples each of ganglionic (blue) and aganglionic colon (green). **(B)** Averaged baseline tone (i.e., isometric force, given in mN) of the circular muscles of all 8 patients, both ganglionic and aganglionic. In addition, the mean value of all eight samples is shown as a horizontal line. No significant difference could be detected between the two groups. **(C)** The administration of EDTA leads to a significant decrease in baseline tone, whereas atropine or vehicle had no effect. **(D)** The administration of EDTA resulted in a complete suppression of any phasic contractions compared to atropine or vehicle.

Following another equilibration period of 60 min (period “d” in Figure 1B), either EDTA or atropine or vehicle or carbachol were applied (period “e” in Figure 1B). Figure 2A1 shows representative recordings of specimens taken from the ganglionic segment of patient #3 to demonstrate the effects of EDTA or atropine. In the presence of 40 mM EDTA, the basal tone dropped markedly by about 40%; and the phasic contractions ceased completely indicating that both forms of spontaneous motility were Ca^2+^-dependent. Importantly, removal of EDTA from the bath fully re-established the spontaneous motility (2nd trace in Figure 2A1). On the contrary, 1 µM atropine had no effect on the ganglionic colon specimen (3rd trace in Figure 2A1).

On the whole, specimens from aganglionic tissue of the same patient (#3) presented with similar spontaneous motility and Ca^2+^ dependence, respectively (Figure 2A2): 40 mM EDTA reduced both basal tone and phasic contractions, while 1 µM atropine failed to affect spontaneous motility. Having a closer look, the basal tone appeared to be smaller in aganglionic than ganglionic tissue just before the application of EDTA or atropine (equivalent to the first gray box in Figures 1C, 2A1,A2, respectively). We therefore averaged the basal tone in each patient, compared all patients in a paired fashion and obtained no significant group differences on the mean basal tone (*P* = 0.228, *n* = 8 vs. *n* = 8, paired *t*-test; Figure 2B). The EDTA traces from patient #3 suggested that the time of onset of the EDTA effect might have been delayed. We also compared this measure in all eight patients, but the onset of EDTA effect was not statistically significant (ganglionic: 3.07 ± 0.77 min; aganglionic: 2.98 ± 0.89 min, *P* = 0.502, paired *t*-test, data not shown).

Next, we aimed to compare directly the Ca^2+^ dependence between ganglionic and aganglionic tissues. To this end, we calculated the percentage change of basal tone and phasic contraction amplitudes, respectively, in response to EDTA or atropine, respectively. Across all eight patients, the population statistics showed significantly reduced basal tone (Figure 2C) and abolished phasic contraction amplitudes (Figure 2D) by EDTA only, yet without revealing any significant difference between ganglionic and aganglionic specimens. Taken together, our experiments at best identified a slightly reduced basal tone in aganglionic tissue compared to ganglionic tissue, but showed a rather weak phenotype of aganglionic circular muscle as long as spontaneous motility is concerned.

### 3.3 Cholinergic responses from ganglionic and aganglionic tissue

**Figure 3:**
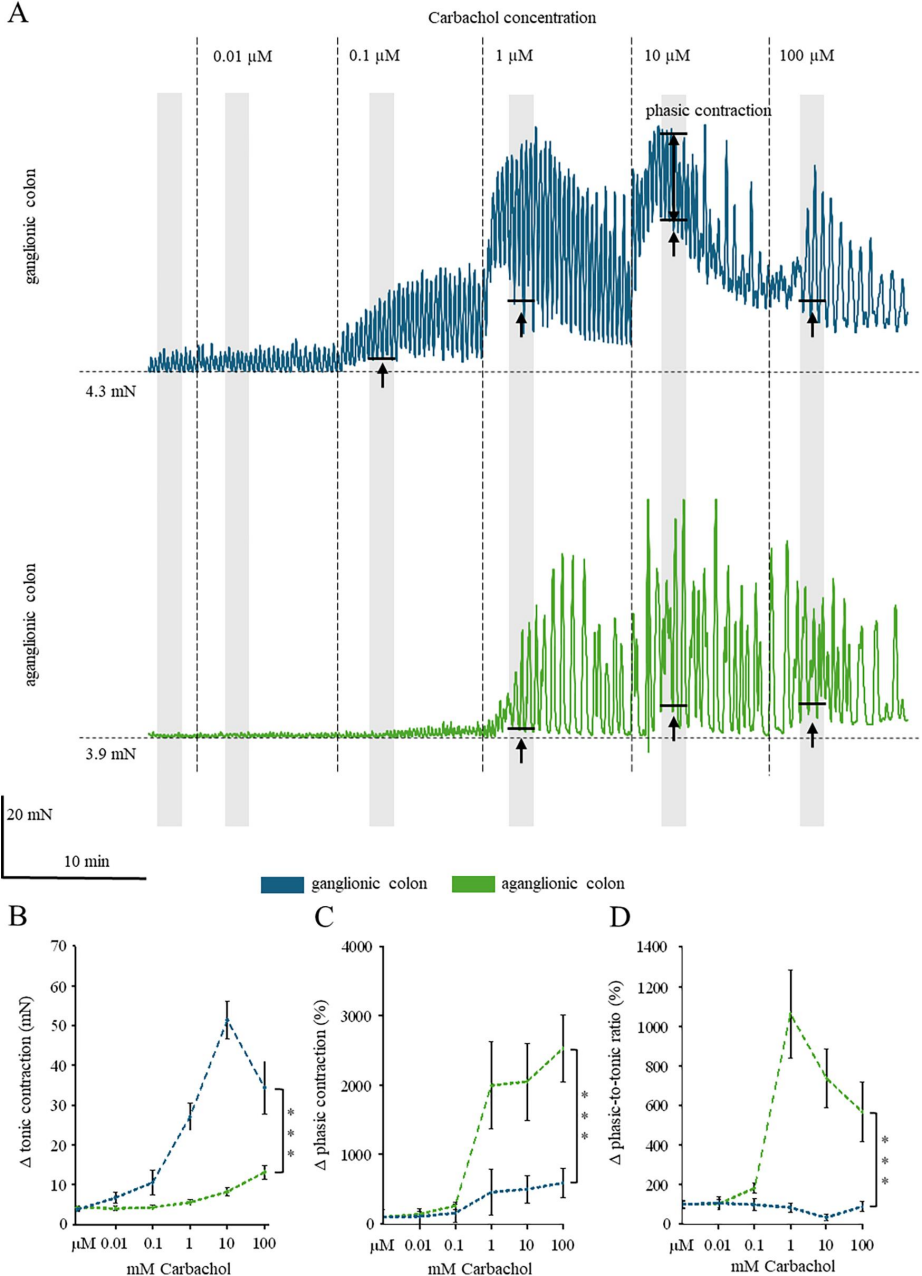
**(A)** Shows carbachol-induced contractions with increasing doses at the ganglionic (blue) and aganglionic colon (green). Every 10 min, a 10-fold increase in dose was achieved. The measurement periods in which basal tone (i.e., isometric force, given in mN) and contraction amplitude were determined are highlighted in gray. The mean values of the basal tone are shown as an example. The color coding is retained for the other diagrams. **(B)** Dose-response curve of the carbachol-induced change in basal tone. There is not only a significantly higher increase in basal tone, but also a shift in the dose-response curve in the aganglionic colon, indicating a decrease in affinity. **(C)** Dose-response curve of contraction amplitudes. The ganglionic colon displays significantly higher contractile amplitudes than the aganglionic colon **(D)** Dose-response curve of the ratio between contractile amplitude and basal tone. It shows a stable ratio in the ganglionic colon, while there is a significant increase in the aganglionic colon.

The second aim of this study was to compare cholinergic responses between ganglionic and aganglionic colon. To this end, we increased the carbachol concentrations (0.01–100 µM) to the tissue. Figure 3A shows representative recordings from patient #4. The specimen from ganglionic colon (blue trace) presented a concentration-dependent increase of both tonic contraction and phasic contraction amplitudes with a saturation effect at 10 µM. In marked contrast, the specimen from the aganglionic colon (green trace) showed a dramatically reduced carbachol-induced tonic contraction, but phasic contractions were still observed. Across all eight patients, we found a half-maximum effect at a concentration of ∼1 µM vs. ∼10 µM for carbachol-induced tonic contraction in ganglionic and aganglionic tissue, respectively (Figure 3B). The carbachol-induced tonic contraction was significantly larger in ganglionic than in aganglionic tissue (*P* < 0.001, two-way ANOVA, Figure 3B). With respect to phasic contraction amplitudes, however, the concentration of the half-maximum effect (∼1 µM) was comparable in both tissue types, but, more importantly, carbachol-induced phasic contractions were significantly enhanced in aganglionic compared to ganglionic tissue (*P* < 0.001, two-way ANOVA, Figure 3C). To measure the maximum achievable contraction by carbachol, we summed the phasic and the tonic contraction. The maximum effect was reduced in aganglionic tissue (49.72 ± 6.01 mN, *n* = 8) compared to ganglionic tissue (70.38 ± 3.51 mN, *n* = 8, *P* < 0.001, paired *t*-test). Thus, phasic contraction amplitudes by far exceeded the tonic contraction in aganglionic tissue. To directly address this issue, we calculated a phasic-to-tonic contraction ratio, which showed a clear concentration-dependent increase in aganglionic, but not in ganglionic tissue (Figure 3D). Further example traces are shown in Supplementary Figure S3.

At the end of the experiment, high KCl concentration was repeated (period “h” in Figure 1B) to demonstrate intact depolarization-induced contraction (ganglionic: 15.2 ± 1.9 mN, *n* = 32; aganglionic: 9.0 ± 1.4 mN, *n* = 32, *P* = 0.522, ANOVA with Holm-Sidak *post-hoc* test), but we still obtained a difference between ganglionic and aganglionic tissue (*P* < 0.001, ANOVA with Holm-Sidak *post-hoc* test).

## 4 Discussion

In the present study, we compared aganglionic and ganglionic circular muscle strips from eight Hirschsprung's disease patients with respect to spontaneous motility and cholinergic responses. First, we found that spontaneous motility was largely comparable including Ca^2+^ dependence and atropine insensitivity. Second, aganglionic specimens showed significantly reduced tonic contraction following cholinergic stimulation, while phasic contractions were preserved.

Spontaneous motility consists of a basal tone as well as phasic contractions riding on this tone; both could be obtained from ganglionic and aganglionic circular muscle. Hence, we conclude that both basal tone and phasic contractions also exist in aganglionic specimens obtained from Hirschsprung's disease bowel. The presence of spontaneous motility consisting of a basal tone and phasic contractions in aganglionic tissue was demonstrated before (16), but without directly comparing ganglionic and aganglionic tissue from the same patients. Our experiments showed that the circular muscle from aganglionic tissue developed a reduced tone compared with ganglionic tissue. When estimating the basal tone, it is important to note that tissue which is inserted into the organ bath at room temperature and eventually rises to body temperature is expected to show an increase of volume and thereby an increase of buoyant force. Hence, one would expect a decrease of isometric force to be recorded rather than an increase. In fact, we found an overall increase of tone during the equilibration time, leading us to believe that the difference between the two tissue types is not an artifact of equilibration. In conclusion, we propose that circular muscle from aganglionic Hirschsprung's disease bowel shows a reduced basal tone, but we agree that this finding will need to be confirmed in a larger sample size before being generalizable. Hence, one has to bear in mind that the relatively small sample size of the current study is a limitation, but it is important to note that current literature only comprises of a few studies that provide functional data on Hirschsprung's disease (19, 26, 27).

Although reduced basal tone might be regarded as a marginal phenotype, it goes along with a reduced propensity to exhibit phasic contractions. Phasic contractions in aganglionic tissue have been demonstrated in the piebald mouse model (28) as well as in the aforementioned study on human aganglionic circular muscle (16), but were absent in aganglionic longitudinal muscle (19). The former human study (16) mentioned that rhythmic contractions were present in 88% of the specimens analyzed. However, aganglionic and ganglionic tissue samples from the same patient were only available in three cases, so the authors could not compare both tissue types with respect to phasic contractions. Here, we directly compared ganglionic and aganglionic specimens in a paired condition and found phasic contractions in 80% of ganglionic and 60% of aganglionic specimens during the equilibration phase. In addition, phasic contractions in aganglionic specimens had a smaller amplitude than those of ganglionic specimens.

Both the basal tone and the phasic contractions of both tissue types were abolished by EDTA, hence were Ca^2+^ dependent. This is consistent with the common concept of smooth muscle contraction following phosphorylation of the 20-kDa myosin light chains (29). However, Ca^2+^-independent pathways have been described and may coexist, e.g., in the esophagus (30). With respect to Hirschsprung disease, at least in the endothelin B receptor-deficient rat model of Hirschsprung disease, the Ca^2+^-independent pathway involving Rho-kinase was found to be upregulated (31). Since in our hands both basal tone and phasic contractions were abolished in EDTA, we suggest that Ca^2+^-independent pathways play a minor role in circular colonic muscle, regardless of whether from ganglionic or aganglionic bowel.

In contrast to EDTA, atropine left spontaneous motility thoroughly unaltered, and—importantly—did so in both tissue types. This finding implies that intrinsic muscarinic receptor activation did not contribute to neither the basal tone nor the phasic contractions and was somewhat unexpected because acetylcholine levels in aganglionic tissue were reported to be three-fold compared to ganglionic tissue (15). One problem is that neurons do not project directly onto muscle cells, as previously assumed, but rather indirectly via ICCs. We are therefore unable to directly determine the direct influence of the reduced ICC on transmission *in vivo*. Here, it has to be mentioned that our experiments cannot rule out nicotinic receptor activation. Indeed, nicotinic receptor staining was increased in human HD tissue, but with particular emphasis on the mucosal layer (32), which was removed in the present study. This also holds true for the reportedly enhanced choline esterase activity in that layer (33). Therefore, we conclude that basal tone and phasic contractions are less likely the result of cholinergic innervation. Rather, we suggest that intramural intrinsic pacemaker mechanisms may govern spontaneous motility, and at least in our hands, there was no difference between aganglionic and ganglionic specimens.

The major finding of the present study was the differential effect of carbachol on tonic and phasic contractions. While the latter were enhanced rather than reduced, tonic contraction was significantly impaired in aganglionic circular muscle compared with ganglionic tissue. Albeit reduced acetylcholine-evoked contraction was observed before in human HD circular muscle (16), our finding further strengthens this notion, because in the aforementioned study, only two out of three paired samples (i.e., aganglionic and ganglionic specimens from the same case) showed a reduced acetylcholine sensitivity. With respect to the mechanisms involved, data from an HD rat model suggested a reduced Cav1.2 transcription, but in that model, both carbachol-induced tonic and phasic contractions were reduced (34), in contrast to our data. Moreover, L-type Ca^2+^ channel currents from patients with HD presented normal activation and inactivation properties and intact sensitivity towards nifedipine and FPL64176 (35). Further studies should therefore address the intracellular signaling cascades following carbachol stimulation and the specificity of intracellular calcium homeostasis. Also, our finding cannot be explained by a simple down-regulation of muscarinic receptors as demonstrated in human HD full-thickness samples (36). Another major question remains regarding the influence on rhythmogenesis. This is still largely unknown in Hirschsprung's disease. Previous studies have shown an insufficient replacement rhythm (27), at least in aganglionosis, but this could not be confirmed in our measurements. However, further experiments are needed to address this issue. The focus should therefore be on the influence of ICCs and, in particular, hERG channels and HCN channels. These were significantly reduced in Hirschsprung's patients (26, 37–39), but play a role in rhythmogenesis in healthy intestines (40). Hence, the mechanisms underlying the differential carbachol effects in aganglionic tissue await further investigation.

When quantifying cholinergic responsivity in the present study, one should bear in mind that we normalized all carbachol-induced responses to the individual basal tone. This, in fact, could lead to an erroneous interpretation since the basal tone in aganglionic specimens was significantly smaller than in ganglionic tissue. Thus, in absolute terms, the impairment of carbachol-induced tonic contractions was even more pronounced and could have been underestimated by the normalization procedure. Phasic contractions, in turn, also had reduced amplitudes, albeit to a much lesser extent than the impaired tonic contraction. Therefore, the differential effect of carbachol on tonic and phasic contractions in aganglionic vs. ganglionic circular muscle appears to be a robust finding.

Since the pathogenesis of short-segment HD, long-segment HD, and TCA has not yet been conclusively clarified, particularly with regard to similarities and differences, it should be taken into account when interpreting the results that our findings may not be applicable to long-segment HD or TCA. One further limitation to be addressed is the fact that the samples originated from a single facility. However, this ensured that all samples were subject to the same methodology in terms of organ removal, preparation of individual samples and measurements in the organ bath.

Normalizing carbachol responses relative to baseline tone could have underestimated the extent of dysfunction in aganglionic tissue. This remains a methodological limitation. However, due to the preparation with preservation of the submucosa, it was not possible to normalize the weight, as the submucosa is significantly wider in aganglionosis than in ganglionosis (41), which prevented normalization.

Finally, our data show that the basic tone of the aganglionic circular musculature appears to be lower than in the ganglionic area. This does not necessarily contradict the propagated stenosis, as the expansion of hollow organs also has an effect according to Laplace's law. This establishes a connection between the inner diameter of hollow organs, the wall thickness, and the opposing pressures. With relation to HD, this means that the musculature itself does not have to build up high tension, as the other parameters of the equation in the narrow intestine already favor the narrowing. Since we measure the muscle *in vitro* in the organ bath, i.e., with the ring being dissected, we cannot measure the effects of Laplace's law. What is new is that, unlike previous studies, we have preserved the submucosa to investigate its possible influence. This should be taken into account when comparing the results with other studies, where the submucosal layer was completely removed mechanically, which could have damaged or removed cells with pacemaker function.

In summary, circular muscle from the aganglionic segment of patients with HD retains spontaneous motility similar to the equivalent tissue from the ganglionic segment. However, specimens from the aganglionic segment showed dramatically reduced tonic contraction following cholinergic stimulation, while phasic contractions were preserved. We propose that performing functional studies on human HD tissue is a valuable tool to help understanding the pathophysiology in aganglionic bowel and, therefore, improve HD management. Although our data are currently promising for understanding pathophysiology, caution is advised due to limitations. Nevertheless, we hope that new pathophysiological findings will influence decision-making in future healthcare.

## Data Availability

The original contributions presented in the study are included in the article/Supplementary Material, further inquiries can be directed to the corresponding author.
