# Supplementary material for: In vitro study of spontaneous motility and cholinergic responses in the human Hirschsprung's disease colon
Source: Front Pediatr. 2025 Dec 1;13:1698220. doi: 10.3389/fped.2025.1698220 (PMC12702922; doi:10.3389/fped.2025.1698220)
Supplement: Supplementary file 1 [file Datasheet1.pdf]

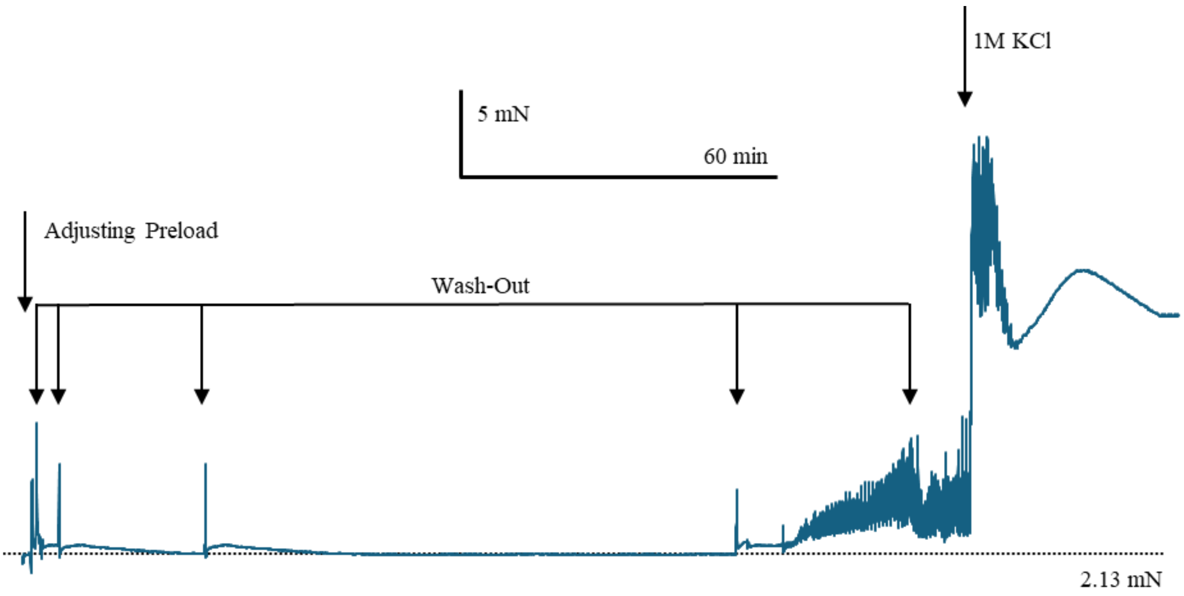

Supplementary figure 1
Illustration of an equilibration period as an example. This lasts 3 hours (see Figure 1, panel B) before stimulation with KCl 1M takes place over 40 minutes. Regular exchanges of the organ bath buffer can be seen. At the beginning, the preload is adjusted several times, as the tissue first has to acclimatise to the environment. During the KCl, a peak in contractions is initially observed before a steady state is reached.

Supplementary figure 2 (Page 2)
Representation of traces from all 8 patients, both ganglionic and aganglionic samples. The different expressions of contraction patterns are clearly visible, whereby it should be noted that all samples exhibit a regular rhythm.

Supplementary figure 3 (Page 3)
Presentation of two additional patients with reactions to carbachol, EDTA, atropine and vehicle in both ganglionic and aganglionic tissue

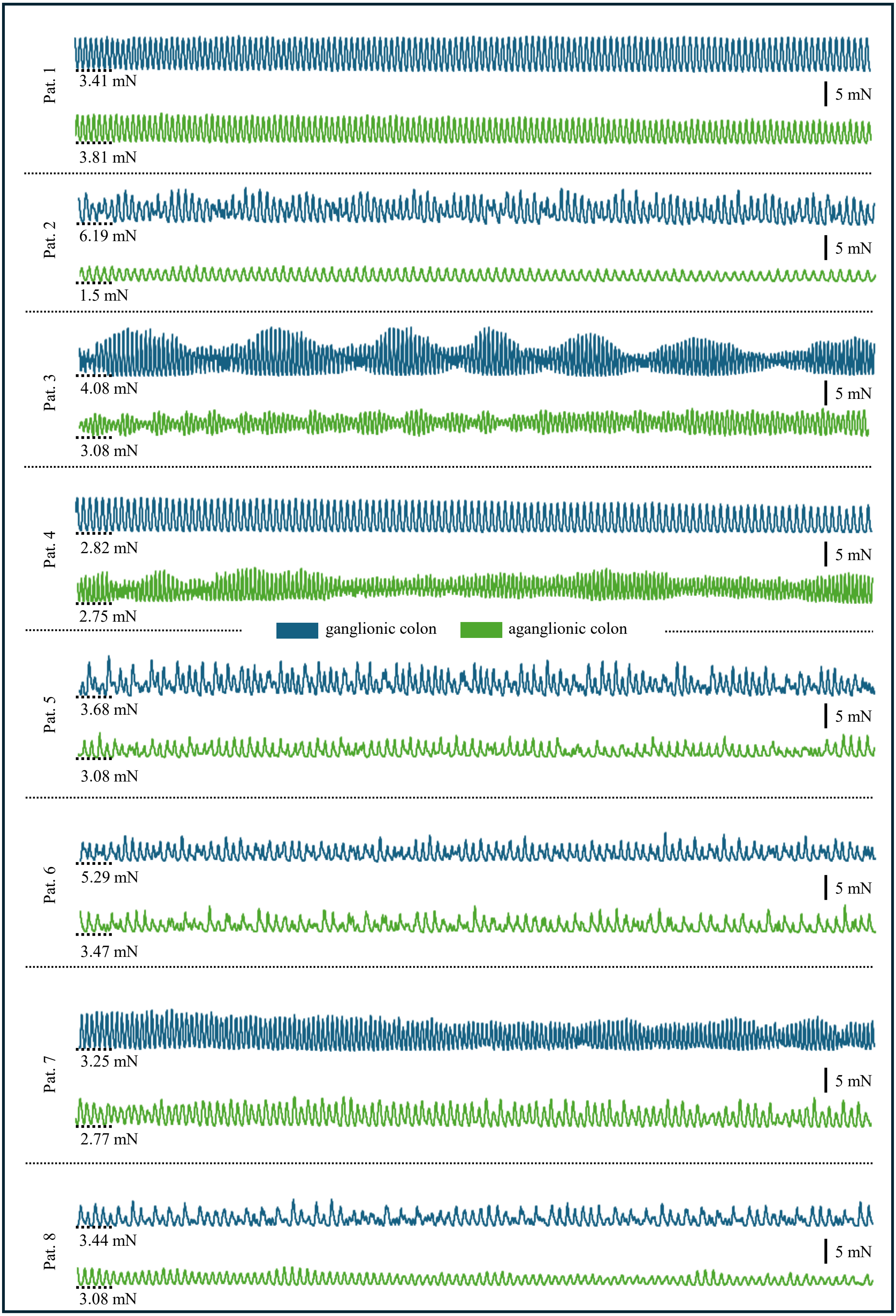

Pat. 1
3.41 mN
5 mN
3.81 mN
Pat. 2
6.19 mN
5 mN
1.5 mN
Pat. 3
4.08 mN
5 mN
3.08 mN
Pat. 4
2.82 mN
5 mN
2.75 mN
ganglionic colon
aganglionic colon
Pat. 5
3.68 mN
5 mN
3.08 mN
Pat. 6
5.29 mN
5 mN
3.47 mN
Pat. 7
3.25 mN
5 mN
2.77 mN
Pat. 8
3.44 mN
5 mN
3.08 mN

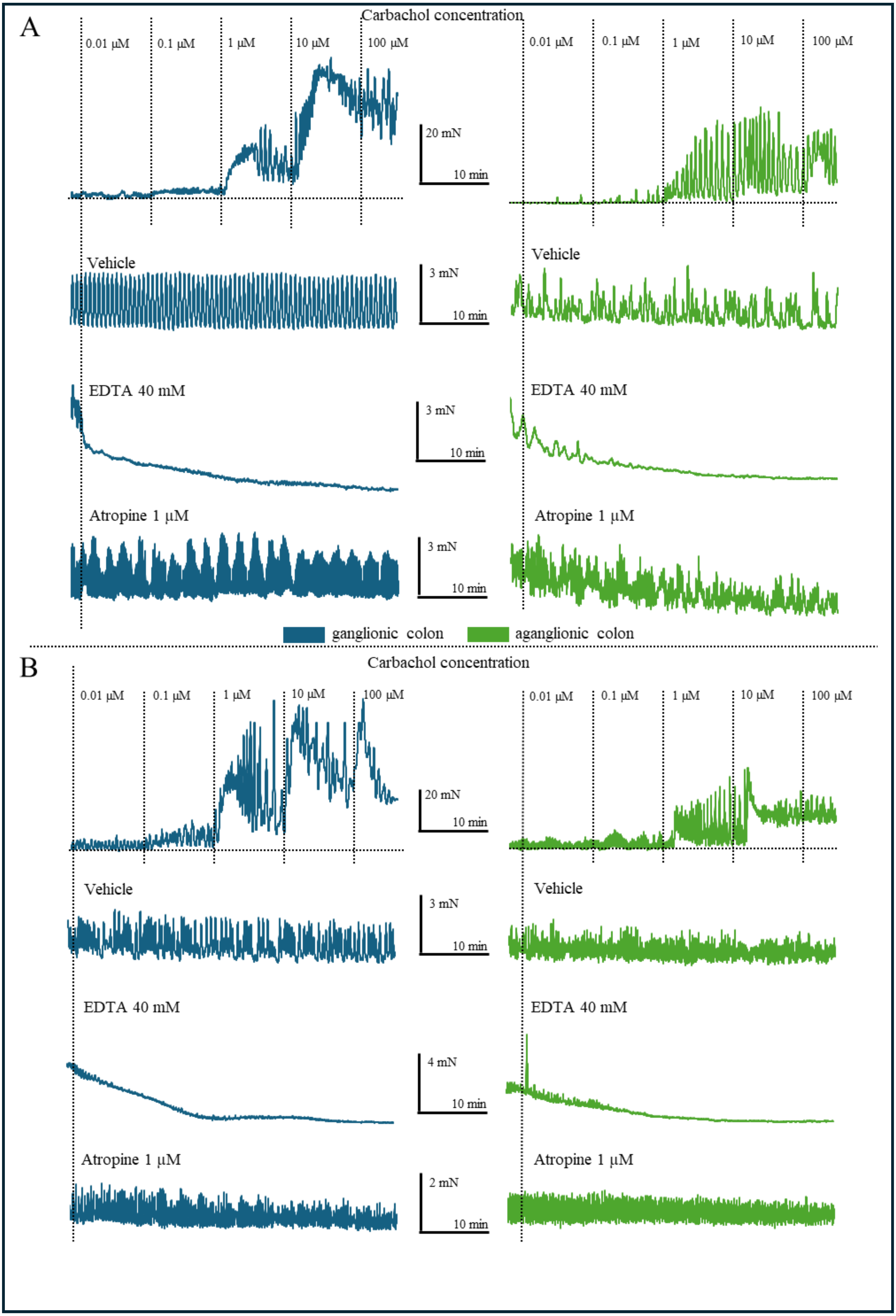
A
Carbachol concentration
0.01 µM
0.1 µM
1 µM
10 µM
100 µM
20 mN
10 min
Vehicle
3 mN
10 min
EDTA 40 mM
3 mN
10 min
Atropine 1 µM
3 mN
10 min
ganglionic colon
aganglionic colon
B
Carbachol concentration
0.01 µM
0.1 µM
1 µM
10 µM
100 µM
20 mN
10 min
Vehicle
3 mN
10 min
EDTA 40 mM
4 mN
10 min
Atropine 1 µM
2 mN
10 min
